# New Analytical Techniques and Applications of Metabolomics and Lipidomics

**DOI:** 10.3390/metabo16010063

**Published:** 2026-01-11

**Authors:** Chunxiu Hu, Xianzhe Shi, Xinyu Liu

**Affiliations:** 1 Metabolomics Subcenter of the National Genomics Data Center, Dalian Institute of Chemical Physics, Chinese Academy of Sciences, Dalian 116023, China; hucx@dicp.ac.cn (C.H.);; 2 Liaoning Province Key Laboratory of Metabolomics, Dalian 116023, China

Metabolomics and lipidomics have emerged as essential tools in systems biology, providing comprehensive insights into small-molecule metabolites and lipids within biological systems [1,2]. The 53rd International Symposium on High-Performance Liquid-Phase Separations and Related Techniques (HPLC 2024 Dalian) served as a dynamic forum for disseminating cutting-edge advances in separation science and its expanding applications. Derived from this conference, this Special Issue of Metabolites captures the innovative spirit and scientific rigor that defined the event. Focused on new analytical methodologies and their applications in metabolomics and lipidomics, the issue includes 14 original research articles and 1 review, collectively illustrating the field’s rapid technical progress and its growing impact across food science, clinical medicine, and fundamental biology.

A central theme emerging from this Issue is the continuous refinement of analytical platforms to achieve greater metabolome coverage and annotation confidence. Wang et al., 2025 (contribution 1) introduced a novel bromine isotope labeling strategy coupled with UHPLC-HRMS, markedly improving the detection and profiling of hydroxyl and amino compounds in sauce-flavored Baijiu. Similarly, Xie et al., 2025 (contribution 2) applied LC-HRMS integrated with Global Natural Products Social Molecular Networking (GNPS) to comprehensively characterize Euryale ferox seeds and monitor processing-induced compositional changes. In a methodological study, Webb et al., 2025 (contribution 3) highlighted how the choice of ultracentrifugation medium (iodixanol vs. KBr) significantly affects the low-molecular-weight metabolome of isolated LDL, an essential consideration for lipoprotein biomarker research. These efforts reflect a growing emphasis on optimizing pre-analytical and analytical workflows to reveal deeper metabolic insights.

The translational potential of metabolomics is strongly evidenced by several studies investigating human pathophysiology. In oncology, Zhou et al., 2025 (contribution 4) uncovered extensive metabolic reprogramming in gastric cancer, identifying altered fatty acid metabolism and TNM-stage-associated subnetworks. Liu et al., 2025 (contribution 5) combined multi-omics data to define glycerolipid metabolism-associated molecular subtypes in pancreatic cancer and proposed ALDH2 as a novel prognostic biomarker. Beyond cancer, Lu et al., 2025 (contribution 6) used integrated metabolomics and lipidomics to demonstrate how chiglitazar ameliorates sepsis-induced acute lung injury by modulating NAD+ and triglyceride homeostasis via the SIRT1/PGC-1α pathway. Zhang et al., 2025 (contribution 7) provided a foundational resource by establishing serum metabolite and lipid reference intervals for a healthy Chinese population, crucial for clinical biomarker discovery. Further demonstrating diagnostic utility, Han et al., 2024 (contribution 8) showed that serum metabolomics can enable early detection and pathogen classification in bloodstream infections, potentially overcoming limitations of traditional culture methods.

This collection also underscores the power of integrated and spatial omics approaches. Yao et al., 2025 (contribution 9) identified plasma metabolites associated with walking ability heterogeneity and decline in older adults, offering clues to metabolic resilience. Liu et al., 2025 (contribution 10) revealed a pathogenic role for polyunsaturated fatty acid imbalances in chronic pancreatitis-induced osteoporosis. Employing spatial resolution, Zhang et al., 2024 (contribution 11) combined single-cell RNA sequencing with spatial metabolomics to map lipid metabolic alterations to specific injured renal cell types in diabetic kidney disease, adding anatomical context to metabolic dysregulation. Such integrative strategies are increasingly vital for unraveling complex physiological and disease processes.

Applications in food and plant sciences are also well represented. Feng et al., 2025 (contribution 12) characterized aroma-active volatiles in different types of Guangnan Dixu tea using HS-SPME-GC-MS and odor activity value analysis. Meng et al., 2024 (contribution 13) identified metabolic biomarkers of freshness in refrigerated vegetables, supporting quality control in the food supply chain. In toxicology, Demicheva et al., 2025 (contribution 14) proposed lipid biomarkers for immunodeficiency states using HPLC-HRMS and bioinformatics.

The Issue concludes with a timely review by Lin et al., 2025 (contribution 15), summarizing the role of bile acids in glucose homeostasis and their therapeutic potential in type 2 diabetes, illustrating how metabolomics informs mechanistic biology and treatment strategies.

In summary, the works presented in this Special Issue reflect the vitality and maturity of metabolomics and lipidomics as disciplines. They highlight not only technological sophistication but also the capacity to generate biologically impactful and clinically actionable knowledge.

## Data Availability

No new data were created or analyzed. Data sharing is not applicable.
